# Preventing Developmental Origins of Cardiovascular Disease: Hydrogen Sulfide as a Potential Target?

**DOI:** 10.3390/antiox10020247

**Published:** 2021-02-05

**Authors:** Chien-Ning Hsu, You-Lin Tain

**Affiliations:** 1 Department of Pharmacy, Kaohsiung Chang Gung Memorial Hospital, Kaohsiung 833, Taiwan; chien_ning_hsu@hotmail.com; 2 School of Pharmacy, Kaohsiung Medical University, Kaohsiung 807, Taiwan; 3 Department of Pediatrics, Kaohsiung Chang Gung Memorial Hospital and Chang Gung University College of Medicine, Kaohsiung 833, Taiwan; 4 Institute for Translational Research in Biomedicine, Kaohsiung Chang Gung Memorial Hospital and Chang Gung University College of Medicine, Kaohsiung 833, Taiwan

**Keywords:** hydrogen sulfide, cardiovascular disease, hypertension, atherosclerosis, cysteine, developmental origins of health and disease (DOHaD), N-acetylcysteine

## Abstract

The cardiovascular system can be programmed by a diversity of early-life insults, leading to cardiovascular disease (CVD) in adulthood. This notion is now termed developmental origins of health and disease (DOHaD). Emerging evidence indicates hydrogen sulfide (H_2_S), a crucial regulator of cardiovascular homeostasis, plays a pathogenetic role in CVD of developmental origins. Conversely, early H_2_S-based interventions have proved beneficial in preventing adult-onset CVD in animal studies via reversing programming processes by so-called reprogramming. The focus of this review will first summarize the current knowledge on H_2_S implicated in cardiovascular programming. This will be followed by supporting evidence for the links between H_2_S signaling and underlying mechanisms of cardiovascular programming, such as oxidative stress, nitric oxide deficiency, dysregulated nutrient-sensing signals, activation of the renin–angiotensin system, and gut microbiota dysbiosis. It will also provide an overview from animal models regarding how H_2_S-based reprogramming interventions, such as precursors of H_2_S and H_2_S donors, may prevent CVD of developmental origins. A better understanding of cardiovascular programming and recent advances in H_2_S-based interventions might provide the answers to bring down the global burden of CVD.

## 1. Introduction

Cardiovascular disease (CVD) is the leading cause of death worldwide, accounting for almost one third of all global deaths [1]. CVD is a cluster of disorders of the heart and blood vessels and is comprised of coronary heart disease, peripheral vascular disease, cerebrovascular disease and other conditions. Although CVD is most common in older adults, atherosclerosis can begin in childhood and progress slowly across the life span [2]. Therefore, reducing the global burden of CVD by identifying children at risk and providing preventive interventions early are extremely important. Noteworthy, CVD can originate from the early stages of life, not only childhood but tracing back into the fetal life. This theory is now termed the developmental origins of health and disease (DOHaD) by observing how a suboptimal environment in utero has an adverse influence on offspring outcomes in later life [3].

The fetal cardiovascular system is vulnerable to adverse early-life environmental insults [4]. Developmental plasticity accommodates morphological and functional changes during organogenesis, leading to endothelial dysfunction, stiffer vascular tree, small coronary arteries, low nephron endowment, and fewer cardiomyocytes, through a process known as cardiovascular programming [4,5,6]. So far, several mechanisms underlying cardiovascular programming have been proposed, like oxidative stress, nitric oxide (NO) deficiency, activation of the renin–angiotensin system (RAS), dysregulated nutrient-sensing signals, and dysbiosis of gut microbiota [4,5,6].

Hydrogen sulfide (H_2_S), the third gasotransmitter, has emerged as a crucial regulator of cardiovascular homeostasis [7,8,9]. H_2_S exerts multifaceted biological functions, including vasodilatation, angiogenesis, antioxidant, anti-inflammation, mitochondria bioenergetics, and antiapoptosis [10,11]. In this regard, H_2_S-releasing drugs have been considered as potential therapeutics for CVD [7,8]. It is noteworthy that the DOHaD concept provides a strategy termed reprogramming to reverse or postpone the programming processes in early life, accordingly protecting offspring against many adult diseases of developmental origins [12]. Emerging evidence suggests that H_2_S can be used as a reprogramming strategy in hypertension of developmental origins [13]. Although H_2_S has been shown to have beneficial effects on CVD [7,8], whether it could serve as a reprogramming intervention for developmental origins of CVD remains largely unclear.

The central aim of this review is to give an overview of H_2_S implicated in cardiovascular programming. The use of H_2_S-based interventions as a reprogramming approach to protect offspring against CVD of developmental origins will be summarized.

Our search strategy was designed to retrieve related literature from PubMed/MEDLINE indexed articles. We used different combinations of search terms: “cardiovascular disease”, “developmental programming”, “DOHaD”, “atherosclerosis”, “heart”, “vascular”, “mother”, “endothelial dysfunction”, “hydrogen sulfide”, “cysteine”, “garlic”, “pregnancy”, “offspring”, “progeny”, “reprogramming”, and “hypertension”. We also used the reference lists of identified articles to find other potential studies. The last search was conducted on 30 December 2020.

## 2. Hydrogen Sulfide in the Cardiovascular System

### 2.1. H_2_S Signaling Pathway

H_2_S, a colorless gas with a characteristic foul odor of rotten eggs, was first identified as an environmental toxin in the 1700s and opened three centuries of research into its biological roles [14]. In the late 1990s, H_2_S was reclassified as the third gaseous signaling molecule, alongside nitric oxide (NO) and carbon monoxide (CO) [10]. Currently, H_2_S is known as a ubiquitous second messenger molecule with important functions in cardiovascular physiology [10,15]. Much of the previous work investigating the actions of H_2_S has been directly focused on incident CVD; however, there is a growing need to better understand the mechanisms and pathways of H_2_S signaling in CVD of developmental origins.

**Figure 1:**
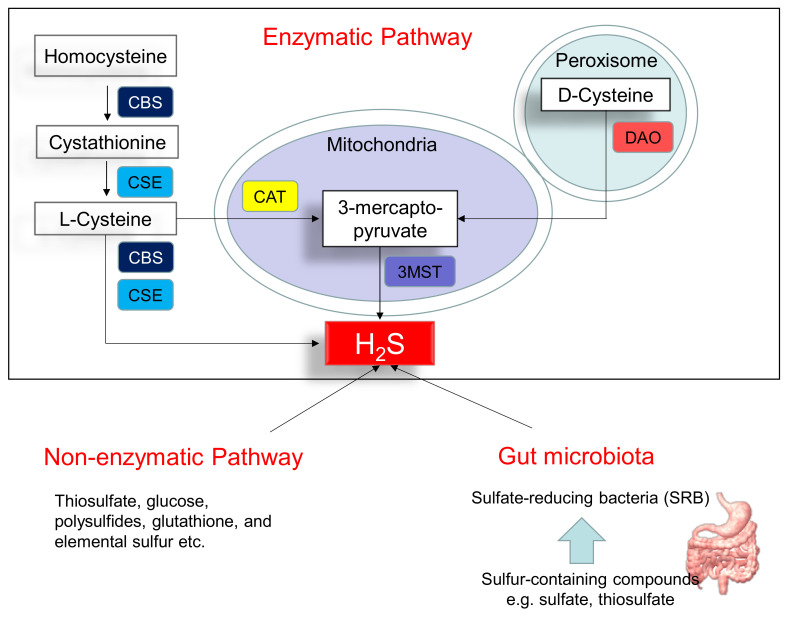
Schematic representation of three major sources of H_2_S: enzymatic pathway, nonenzymatic pathway, and bacterial origins. Cystathionine β-synthase (CBS) catalyzes homocysteine to produce Cystathionine. Cystathionine γ-lyase (CSE) catalyzes cystathionine to form l-cysteine or l-cysteine to generate H_2_S. 3-Mercaptopyruvate sulfurtransferase (3MST) produces H_2_S from 3-mercaptopyruvate, which is generated by cysteine aminotransferase (CAT) and d-amino acid oxidase (DAO) from l-cysteine and d-cysteine, respectively. Another source of endogenous H_2_S is coming from nonenzymatic processes. The other source of H_2_S is derived from gut microbes, mainly by the sulfate-reducing bacteria (SRB).

Figure 1 illustrates three major pathways of H_2_S synthesis, including enzymatic pathway, nonenzymatic pathway, and bacteria origins. Three enzymes have been identified to enzymatically generate H_2_S, cystathionine β-synthase (CBS), cystathionine γ-lyase (CSE), and 3-mercaptopyruvate sulphurtransferase (3MST) [10]. CBS and CSE are cytosolic enzymes, but 3-MST is mainly existing in the mitochondria. l-cysteine is the principal substrate for both CBS and CSE to generate H_2_S. CBS and CSE can also produce H_2_S using other substrates. Homocysteine can be catalyzed by CBS to generate cystathionine, followed by CSE to produce l-cysteine. All of the above-mentioned H_2_S-generating enzymes are expressed in the heart and blood vessels [10,16]. In an alternative pathway, 3-mercaptopyruvate, the substrate for 3-MST to produce H_2_S, is provided by cysteine aminotransferase (CAT) and D-amino acid oxidase (DAO). In the peroxisome, d-cysteine can be catabolized by DAO to generate H_2_S [17]. Besides the enzymatic pathway, H_2_S can be nonenzymatically produced through thiosulfate, glucose, polysulfides, glutathione, and elemental sulfur.

Another source of H_2_S is coming from the gut microbiota. Approximately fifty percent of fecal H_2_S is derived from bacteria. In the gut, sulfate-reducing bacteria (SRB) obtain energy from the oxidation of organic compounds, reducing sulfate to H_2_S. *Desulfovibrio* account for 66% of all SRB in the human colon [18]. Other gut bacteria may also produce H_2_S by sulfite reduction, including species *E. coli, Enterobacter, Salmonella, Klebsiella, Bacillus, Corynebacterium, Staphylococcus,* and *Rhodococcus* [19]. Conversely, sulfur-oxidizing bacteria (SOB) reduces H_2_S via sulfur oxidation. The SOB members include genera *Acidithiobacillus*, *Bacillus*, *Paracoccus*, *Pseudomonas*, and *Xanthobacter*. In the gut, a huge quantity of H_2_S is oxidized by colonocytes to thiosulfate. The existence of thiosulfate in cecal venous blood not only reflects the detoxification of H_2_S but also the recycling of H_2_S.

In the circulation and tissues, free H_2_S can be scavenged and stored in the bound-sulfate and sulfane sulfur pools. Methylation and oxidation are two major mechanisms of H_2_S metabolism. H_2_S can be excreted in urine and flatus as free sulfate, free sulfide or thiosulfate.

### 2.2. The Role of H_2_S in the Pathophysiology of CVD

Multiple lines of evidence indicate that H_2_S plays a crucial role in the pathogenesis of CVD. The first are reports on knockout mice lacking genes encoding for CSE, CBS, and 3-MST. CSE is the most relevant H_2_S-producing enzyme in the cardiovascular system. Mutant mice lacking CSE had decreased H_2_S levels in the serum, heart, vessels, and other tissues [20]. CSE knockout mice displayed hypertension, endothelial dysfunction, and accelerated atherosclerosis [20,21]. CBS-deficient mice developed endothelial dysfunction [22] and cerebral vascular dysfunction [23]. 3-MST knockout mice developed hypertension and cardiac hypertrophy [17]. Second, are observations that impaired H_2_S-generating pathways were found in CVDs, including atherosclerosis [24], coronary artery disease [25], stroke [26], and peripheral vascular disease [15].

Third, are studies of protein S-sulfhydration, a vital post-translational modification induced by H_2_S [9]. S-sulfhydration usually increases the reactivity of target proteins via formation of a cysteine persulfide to target proteins [9]. H_2_S is able to S-sulfhydrate Kelch-like ECH associated protein 1 (Keap1), specificity protein-1 (SP-1), nuclear factor kappa-B (NF-κB) and interferon regulatory factor-1 (IRF-1) to regulate target gene transcription, which is crucial for the regulation of endothelial phenotypes, myocardial hypertrophy, mitochondrial biogenesis, oxidative stress, apoptosis and inflammation [9].

Fourth, several H_2_S-releasing drugs have demonstrated considerable promise for beneficial effects against CVDs in various animal models [7,8]. As reviewed elsewhere [7], several cytoprotective actions of H_2_S have been reported in the heart and vasculature. In the heart, the protective effects of H_2_S signaling was related to anti-inflammation, antiapoptosis, reduction of oxidative stress, and antifibrosis that leads to cardiac remodeling and functional improvements. In the vessels, H_2_S signaling can preserve endothelial NO synthase (eNOS)-derived NO production, while reducing oxidative stress, inflammation, fibrosis, and smooth muscle cell proliferation.

### 2.3. H_2_S Signaling in Various CVDs

Endothelial dysfunctions are associated with various CVDs, including hypertension, atherosclerosis, myocardial infarction, and the cardiovascular complications of diabetes. H_2_S can prime endothelial cells toward angiogenesis and contribute to relax vascular smooth muscle cells, and thereby reducing BP [27]. A deficit in H_2_S homeostasis is involved in the pathogenesis of endothelial dysfunction, while the application of H_2_S-releasing drugs to increase endogenous H_2_S level can restore endothelial function and antagonize the progression of CVDs.

Hypertension is a key risk factor for multiple CVDs. Like NO, H_2_S is a vasodilator. H_2_S has been reported to relax various blood vessels, such as the rat thoracic aorta, portal vein, and peripheral resistance vessels [28,29,30]. The involvement of H_2_S deficiency in hypertension has been examined in various animal models of hypertension, including the spontaneously hypertensive rat (SHR) [31], the renovascular hypertensive model [32], Dahl salt-sensitive rats [33], and NO-deficient rats [34]. Conversely, several prior studies have shown the beneficial effects of exogenous and endogenous H_2_S on hypertension, as reviewed elsewhere [35]. However, little is known about whether these H_2_S-based therapies could be used as reprogramming interventions perinatally to reduce the vulnerability to developing cardiovascular programming in offspring.

ApoE knockout mice developed advanced atherosclerosis related to a decreased plasma H_2_S level and vascular CSE expression/activity, suggesting disturbance of the vascular CSE/H2S pathway plays a role in the pathogenesis of atherosclerosis [36]. Additionally, a reduction in circulating H_2_S has also been noted in diabetic animal models and diabetic patients [37]. Conversely, H2S therapy proved beneficial in diabetes-accelerated atherosclerosis in diabetic mice [38]. In a rat model of myocardial ischemia–reperfusion (I/R), pharmacologic inhibition of CSE resulted in an increase in infarct size, whereas H_2_S replacement displayed myocardial protection [39]. Likewise, cardiac-specific overexpression of CSE in mice protects against myocardial I/R injury [40]. Summarizing, in clinical and preclinical studies of various CVDs, endogenous H_2_S production is diminished in these pathological conditions and H_2_S deficiency contributes to the progression of disease [7].

## 3. Evidence from Human Studies for Cardiovascular Programming

Important support for cardiovascular programming came from epidemiological reports following birth cohorts in the severe famines (Saint Petersburg 1941–1944; Dutch 1944–1945; Biafra 1967–1971) [41,42,43]. These observations revealed that exposure to undernutrition in early life induced a cluster of metabolic syndrome-related phenotypes such as hypertension, dyslipidemia, obesity, type 2 diabetes, and cardiovascular morbidity, all risks factor for coronary artery disease. Together with undernutrition, other environmental influences that can program later CVD have also been reviewed elsewhere [4,5,6]. These influences include maternal overnutrition, maternal smoking, maternal illness, and exposure to medication or environmental toxins. Fetal overnutrition because of maternal diabetes or obesity is related to offspring’s type 2 diabetes and obesity, both risk factors for CVD [44]. There is a positive association of maternal prenatal smoking with child adiposity and high BP [45]. Another report showed an association between maternal bisphenol A exposure and cardiometabolic traits in childhood [46]. In twins, the association between birth weight and BP is described in infants [47] and the lower bodyweight twins are prone to die from ischemic heart disease [48]. Moreover, several other perinatal risks affecting BP and cardiometabolic outcome in offspring have been identified, like low vitamin D intake [49], gestational hypertension [50], short-term breastfeeding [51], and excessive postnatal weight gain [52].

Although the influence of fetal life for future cardiovascular health has been evidenced by various epidemiologic human studies, it is almost impossible to test prospectively for critical developmental windows in humans. Moreover, nearly all cohort studies involve offspring who have not yet reached middle age and defined cardiovascular endpoints. Therefore, it is difficult to establish direct cause-and-effect relationships between particular environmental insults and later clinical cardiovascular outcomes in these cohort studies. Accordingly, it would be logical to use animal models to test our knowledge, for which a developmental window is decisive for cardiovascular programming, to identify how types of early life insults may program cardiovascular phenotypes, and what reprogramming intervention can be applied.

**Figure 2:**
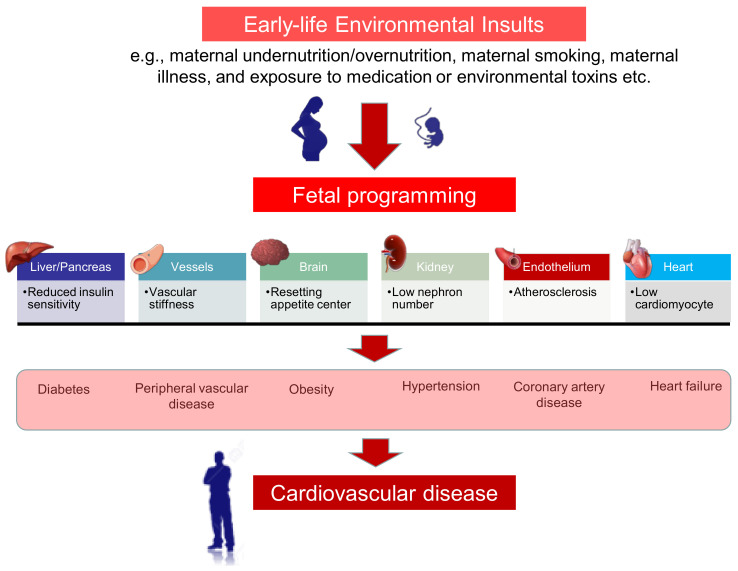
Schematic illustration of links between maternal insults, fetal programming, and cardiovascular disease of developmental origins. A wide range of early-life environmental factors can influence fetal programming, include maternal undernutrition/overnutrition, maternal smoking, maternal illness, and exposure to medication or environmental toxins. During organogenesis, these maternal insults induce morphological and functional changes in different organ systems, such as liver, pancreas, heart, vessels, brain, kidney, and endothelium. Consequently, cardiovascular programming causes a cluster of phenotypes such as diabetes, hypertension, obesity, dyslipidemia, type 2 diabetes, and cardiovascular morbidity, all risks factor for cardiovascular disease.

We propose a schema for summarizing the links between early-life insults, fetal programming, and the programming processes in different organ systems that are involved in the developmental programming of CVD, which is presented in Figure 2.

## 4. Common Mechanisms Link H_2_S to Cardiovascular Programming

Despite a wide range of early-life environmental factors related to CVD in later life, current evidence suggests that there may be common molecular mechanisms underlying cardiovascular programming. Although the complete mechanisms remain inconclusive, animal models have provided important information on particular pathways including oxidative stress, NO, RAS, nutrient-sensing signals, and gut microbiota dysbiosis [4,5,6]. Remarkably, these extensive animal experiments have shown interactions between H_2_S signaling pathway and the abovementioned mechanisms. We will discuss each of these mechanisms in turn.

### 4.1. Oxidative Stress

The role of oxidative stress in the onset and progression of atherosclerosis and CVD has been widely studied [53,54]. Oxidative stress reflects an imbalance between oxidants (e.g., reactive oxygen species (ROS)) and antioxidants in favor of the first. Owing to its low antioxidant capacity, the developing fetus is extremely vulnerable to oxidative damage [55]. As we reviewed elsewhere [6], many maternal insults have been described to induce cardiovascular programming associated with oxidative stress, including undernutrition [56], preeclampsia [57], maternal diabetes [58], maternal exposure to nicotine or ethanol [59,60], maternal high-fat intake [61], and prenatal glucocorticoid or hypoxia exposure [62,63]. Oxidative damage in the heart, kidney, and blood vessels is well-known for its contribution to organ dysfunction and CVD. In the prenatal dexamethasone plus postnatal high-fat diet model, high-fat intake caused hypertension in adult offspring coinciding with reduced renal CBS and 3MST protein levels [62].

In contrast, a reprogramming strategy aimed at the reduction of oxidative stress by the use of perinatal antioxidants has been applied in animal models to prevent adult disease of developmental origins [64]. H_2_S has an antioxidant property, by which it is able to scavenge ROS, increase antioxidant glutathione, and activate nuclear factor E2-related factor 2 (Nrf2), a transcription factor for protection against oxidative stress [9,10,11]. Additionally, N-acetylcysteine (NAC), a precursor for H_2_S synthesis, has been reported to reprogram hypertension in animal models of developmental hypertension, including suramin-induced preeclampsia [57], and prenatal dexamethasone plus postnatal high-fat diet [62].

All together, these observations reveal that H_2_S might counterbalance oxidative stress to protect offspring against cardiovascular programming. Nevertheless, whether the anti-oxidative ability of H_2_S might be interconnected with other mechanisms to prevent CVD of developmental origins remains to be elucidated.

### 4.2. NO Deficiency

Endothelial dysfunction, mainly characterized by NO deficiency, is the initial event in the development of CVD [65]. NO deficiency in CVD is mainly due to L-arginine deficiency (the substrate for NOS), decreased abundance and/or activity of NOS, inactivation of NO under oxidative stress, and increased asymmetric dimethylarginine (ADMA, an endogenous NOS inhibitor) [66,67].

Abundant evidence indicates that the impaired ADMA/NO pathway contributes to the pathogenesis of cardiovascular programming. First, ADMA competes with L-arginine to inhibit NO production [68] and its increase is involved in coronary artery disease [69], congenital heart disease [70], type 2 diabetes [71], stroke [72], obesity [73], and peripheral arterial occlusive disease [74]. Second, gestational NO depletion induced by N^G^-nitro-L-arginine-methyl ester (L-NAME, an inhibitor of NOS) causes cardiovascular programming in adult offspring, characterized as endothelial dysfunction, hypertension, defect of carotid artery, and cardiac hypotrophy [75,76]. Third, reprogramming effects of therapeutic strategies targeting the ADMA/NO pathway to prevent the developmental programming of hypertension have been reported in various animal models [76,77,78,79].

On the other hand, growing evidence supports H_2_S and NO can affect not only the generation of each other but also the further downstream signaling pathway [80]. In the cardiovascular system, H_2_S and NO display some similar functions like regulation of vascular tone, stimulation of endothelial cell angiogenesis and protection against cardiac injury [80]. H_2_S has been reported to increase NO bioavailability via activation of eNOS through the Akt pathway or calcium release [81,82], enhancing eNOS activity by S-sulfhydration [9], decreased cGMP degradation by inhibiting phosphodiesterase activity [83], and reduction of nitrite [84]. Although there is plenty of evidence pointing towards their impacts on cardiovascular programming, much work remains still to be done to investigate the cross-talk between H_2_S and NO.

### 4.3. Renin–Angiotensin System

The RAS is a major hormone cascade involved in the cardiovascular system [85]. There are two pathways in the RAS system: classical and counter-regulatory pathways. The classical RAS is mainly made up of angiotensin-converting enzyme (ACE), angiotensin (Ang) II, and angiotensin type 1 receptor (AT1R). Under pathophysiological conditions, the classical RAS can be activated to trigger inflammation and structural remodeling, thus promoting cardiac and vascular damage [86]. While the ACE2–angiotensin (1–7)–Mas receptor pathway is termed counter-regulatory RAS to counterbalance the detrimental effects of Ang II signaling.

Both pathways have been implicated in fetal programming [87,88]. The classical RAS expression is reduced at birth, but returns to normal level with age [89]. Under pathophysiological conditions where this normalization overcompensates, consequently fetal programming activates the classical RAS, leading to hypertension in later life [89]. On the other hand, early blockade of the classical RAS can prevent developmental origins of hypertension [90,91]. These findings provide support for the view that RAS plays an important role in cardiovascular programming.

Low levels of H_2_S and the downregulation of its producing enzymes were reported in hypertensive models with activation of the classical RAS [92,93]. Conversely, the protective role of H_2_S against hypertension coincided with downregulating RAS-related mRNA expression [94], reducing AT1R protein level [95], and suppressing renin release [93]. Nevertheless, the detailed mechanisms underlying the modulation of RAS components by H_2_S contributing to the protection of CVD of developmental origins await further investigation.

### 4.4. Nutrient-Sensing Signals

Maternal nutritional status governs fetal growth and development by means of nutrient-sensing signals. Imbalanced maternal nutrition can disturb nutrient-sensing signals, leading to fetal programming and adverse cardiometabolic outcomes [96]. In the cardiovascular system, peroxisome proliferator-activated receptors (PPARs), cyclic adenosine monophosphate (AMP)-activated protein kinase (AMPK), silent information regulator transcript (SIRT), and PPARγ coactivator-1α (PGC-1α) are known nutrient-sensing signals [97]. These signals are involved in the pathogenesis in CVD, which have been reviewed extensively elsewhere [98,99].

Our prior research revealed that AMPK activation prevents the development of hypertension programmed via regulation of nutrient-sensing signals in various models of developmental hypertension, including high-fat diet [100], high-fructose diet [101], L-NAME plus postweaning high-fructose diet [102], and prenatal dexamethasone exposure plus postweaning high-fat diet [103]. Additionally, PPARs govern the expression of specific sets of target genes involved in hypertension of developmental origins [104], which can be driven by maternal nutritional insults.

Besides, AMPK, SIRT1, and PGC-1α can mediate autophagy, a self-degradative process that promotes proteolytic degradation of cytosolic components at the lysosome [105]. AMPK can induce mitochondrial biogenesis by activating the PGC-1α, either directly or through the SIRT1 [106]. Since autophagy contributes to the homeostasis in most cells of cardiovascular origin (e.g., cardiomyocytes, endothelial cells, and arterial smooth muscle cells) and the development of CVD [107,108], early interventions by AMPK activators or PPAR modulators have been considered as a potential reprogramming strategy against CVD of developmental origins [109].

AMPK and SIRT1 are considered as main mediators of H_2_S-associated cardiovascular beneficial effects [109]. Activation of AMPK and mediation of autophagy participate in the H_2_S-induced cytoprotective effect [110]. The cardioprotection of H_2_S is associated with AMPK phosphorylation and alleviation of autophagy in a myocardial ischemia mice model [111]. In another study, administration of exogenous H_2_S could inhibit the excessive autophagy of vascular endothelial cells by regulating the AMPK signaling pathway [112]. Moreover, H_2_S can promote SIRT1 activity to mediate angiogenesis [113]. Another report showed the protective effects of H_2_S against ischemia/reperfusion (I/R) injury are related to the activation of SIRT1/PGC-1α in a rat I/R model [114]. These observations demonstrate that the interplay between H_2_S and nutrient-sensing signals are implicated in CVD of developmental origins.

### 4.5. Gut Microbiota Dysbiosis

The gut bacteria can affect the control of the cardiovascular system via two pathways [115]. First, gut bacteria and/or their metabolites can stimulate the enteric afferent sensory fibers, consequently driving the brainstem cardiovascular centers. Second, gut microbiota-derived metabolites are able to enter into the bloodstream and affect the function of the cardiovascular system.

Adverse environmental conditions occurring early in life can alter the microbial composition of the gut, leading to many adult diseases like CVD [116,117]. So far, the adverse effects of gut microbiota dysbiosis on atherosclerosis, myocardial infarction, arrhythmia, and heart failure have been established, as reviewed elsewhere [103]. Several mechanisms underlying gut microbiota dysbiosis have been linked to CVD, including alterations of short-chain fatty acids (SCFA) and tryptophan-derived metabolites, increases of trimethylamine-N-oxide (TMAO), activation of the RAS, and inhibition of NO as well as H_2_S [118,119,120].

Conversely, approaches including probiotics, prebiotics, postbiotics (e.g., SCFAs), or microbial inhibitors that target specific pathways (e.g., TMAO), have shown beneficial cardiovascular effects [121]. Our previous research demonstrated that supplementation with prebiotic inulin, probiotics *Lactobacillus casei*, or postbiotics acetate during pregnancy and lactation can protect adult offspring against hypertension programmed by a variety of maternal insults [122,123,124].

Although gut bacteria-derived H_2_S has been reported to display BP-lowering effect [125], there is still limited information on the role of microbes-derived H_2_S on cardiovascular programming. Recently, data obtained from our laboratory demonstrated that maternal NAC therapy that protected male SHR offspring against hypertension was linked to increased fecal concentrations of H_2_S and thiosulfate, augmentation of H_2_S-producing pathway in the kidneys, and alterations of gut microbiota [126]. As thiosulfate is a metabolite of H_2_S and also an index of the sulfide pool [127], our results suggest that targeting microbe-derived H_2_S might be a potential approach to prevent hypertension and deserves further evaluation. Another study reported that a high-fat diet caused hypertension in adult offspring and was associated with reduced plasma and fetal H_2_S levels, renal H_2_S-releasing activity, and a decrease in α-diversity in the gut microbiota [128]. Conversely, garlic oil therapy in pregnancy and lactation protected adult offspring against hypertension, which was related to increased mRNA abundance and activity of H_2_S-generating enzymes in offspring kidneys as well as increased microbial richness and microbial diversity [128].

Overall, these findings establish a close connection between H_2_S and other important mechanisms involved in cardiovascular programming. Although there is emerging evidence for crosstalk between H_2_S and particular mechanisms related to CVD of developmental origins, more research is required to gain insight into how H_2_S may play an essential role in mediating other mechanisms, to develop a specific strategy to reduce their impact on developmental programming of CVD.

**Figure 3:**
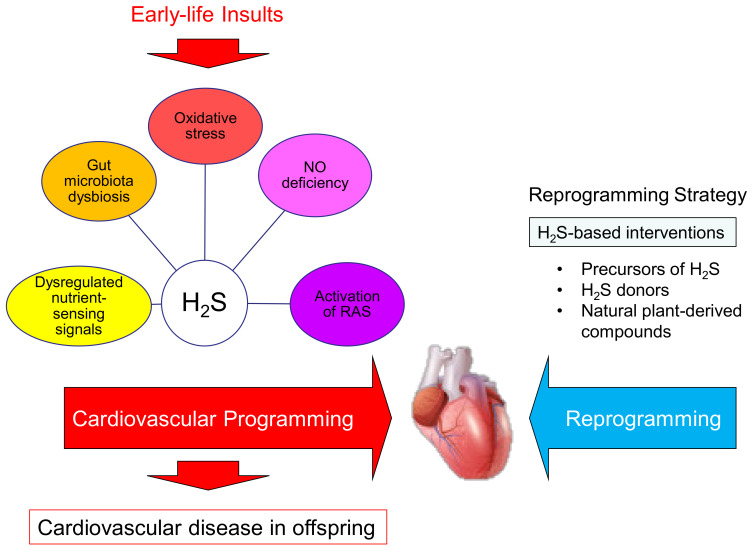
Schema outlining the cardiovascular programming versus reprogramming interventions. Maternal insults can induce cardiovascular programming, consequently leading to cardiovascular disease in adulthood. Hydrogen sulfide (H_2_S) interconnects with other mechanisms and plays a key role in the pathogenesis of cardiovascular programming. These mechanisms include oxidative stress, nitric oxide (NO) deficiency, activation of the renin–angiotensin system (RAS), dysregulated nutrient-sensing signals, and gut microbiota dysbiosis. Conversely, early H_2_S-based interventions may reverse or delay programmed processes to prevent cardiovascular disease of developmental origins by so-called reprogramming.

A summary of the links between H_2_S and other mechanisms implicated in cardiovascular reprogramming by H_2_S-based interventions to prevent developmental programming of cardiovascular disease is depicted in Figure 3.

## 5. H_2_S-Based Reprogramming Intervention

In the past decade, there has been heightened enthusiasm for the development H_2_S-based agents as potential therapeutics [7,8]. Sulfide salts, such as sodium sulfide and sodium hydrosulfide, represent the first class of H_2_S donors [129]. Sulfide salts provide direct and prompt release of free H_2_S. Naturally occurring H_2_S donors derived from garlic and onions generate free H_2_S at a slower rate than sulfide salts [130]. Earlier on, GYY4137 was developed in 2008 as one of the first slow-releasing H_2_S donors [131], and its slow H_2_S-releasing profile better mimics physiological H_2_S production. However, the poor pharmacological properties of sulfide salts and naturally occurring H_2_S donors warrant the need for further development of novel, synthetic H_2_S donors like SG-1002 [132]. Additionally, thiol-activated H_2_S donors (e.g., acyl perthiol donors) [133], pH-controlled H_2_S donors (e.g., JK donors) [134], and enzyme-dependent H_2_S donors [135], have been designed and shown cardiovascular benefits. Although novel H_2_S-donating agents are designed and tested in preclinical models of CVD, few of them have been examined in CVD of developmental origins.

Here, we show Table 1 that summarizes studies documenting H_2_S-based reprogramming interventions in animal models of cardiovascular programming, restricting interventions to critical periods during early development [52,57,72,85,114,136,137,138,139,140]. In the current review, we only considered studies reporting offspring outcomes starting from childhood.

As shown in Table 1, rats have been the dominant animal species used. Various developmental programming models have been studied, including the genetic hypertension model [93,136], suramin-induced preeclampsia model [57], N^G^-nitro-l-arginine-methyl-ester (L-NAME) induced preeclampsia model [76], prenatal dexamethasone and postnatal high-fat diet [62], maternal hypertension [126], high-fat diet [128], maternal nicotine exposure [137,138], and maternal renovascular hypertension model [139,140]. The major adverse cardiovascular outcome is hypertension [57,62,76,93,126,128,139,140], followed by myocardial ischemia-reperfusion injury [137,138] and sympathetic activation [139,140]. The reprogramming effects of H_2_S-based therapies have been reported in rats ranging from 12 week to 8 months of age, which is roughly equivalent to human ages from young to middle adulthood. Available H_2_S-based treatment modalities used as reprogramming interventions include l-cysteine, d-cysteine, NAC, sodium hydrosulfide (NaHS), and garlic.

### 5.1. Precursors of H_2_S

l-cysteine is a sulfur-containing amino acid. Besides, l-cysteine is a component of glutathione, a potent antioxidant in our body. As it is the substrate for H_2_S, l-cysteine supplementation is a way to produce endogenous H_2_S in experimental studies. Since H_2_S and glutathione are closely linked to BP regulation [13,141], l-cysteine has shown an antihypertensive effect [142]. NAC, a stable cysteine analog, has shown beneficial effects for hypertension and CVD in human and experimental studies [143,144]. Another H_2_S precursor, d-cysteine, is nutritionally antagonistic, and, hence, has received less attention [145]. Although one previous report indicating d-cysteine supplementation protects against ischemia/reperfusion injury in the kidney [136], its effect in the heart and vessels remains unknown.

We previously presented that high salt-treated SHRs supplemented with d- or l-cysteine between four and six weeks of age were protected against hypertension and kidney injury at 12 weeks old [136]. Although one study reported that the d-cysteine pathway has an 80-fold greater H_2_S-releasing activity compared to the l-cysteine pathway [146], our results demonstrated that their beneficial effects on BP are comparable. Similarly, early NAC therapy, starting at four weeks of age, has proved beneficial for hypertension in SHRs [147]. As such, the use of NAC therapy in pregnancy and lactation has been shown to have benefits for hypertension of developmental origins in several animal models, including suramin-induced preeclampsia [57], prenatal dexamethasone and postnatal high-fat diet [62], L-NAME-induced preeclampsia [76], and maternal hypertension [126]. Although several H_2_S precursors in response to various insults that have been studied displayed protection against hypertension, there remains a lack of data regarding other cardiovascular benefits.

### 5.2. H_2_S Donors

Inorganic sulfide salts such as sodium hydrosulfide (NaHS) and sodium sulfide (Na_2_S) are the most widely used H_2_S donors to evaluate the therapeutic potential of exogenous H_2_S [7,8]. NaHS has demonstrated protective effects against hypertension in various animal models, including NO-deficient rats [33], Dahl salt-sensitive rats [32], Ang II-infused mice [91], and SHR [148]. Likewise, Na_2_S has shown an antihypertensive effect in SHR [149].

Table 1 shows NaHS therapy between four and six weeks of age prevented hypertension in adult SHRs at 12 weeks of age [93]. In NaHS-treated SHR, NaHS-prevented hypertension coincided with increased H_2_S production and glutathione level [93]. These findings support the notion that H_2_S prodrug may function not only as a source of H_2_S but also as precursors of glutathione, a potent antioxidant in the body [10]. Another report showed that maternal NaHS therapy protects adult offspring against hypertension in a renovascular hypertension model [139]. Additionally, using the same model, maternal NaHS therapy benefits on cardiovascular outcome prevented sympathetic activation [140]. The beneficial effect of NaHS on BP may also be attributed to the reduced expression of angiotensin II type 1 receptor (AT1R)/ROS/inflammation pathway in the brain.

As we mentioned earlier, inorganic sulfide salts induce a rapid but short-lived increase of H_2_S to supraphysiological concentrations. Thus, several organic slow-releasing H_2_S donors have been synthesized to overcome this limitation [7,8]. Although GYY4137 showed a protective effect against hypertension in a CSE inhibition-induced preeclampsia model as well as a L-NAME-treated SHR model [150,151], none of the organic slow-releasing H_2_S donors have yet been tested in terms of their reprogramming effects on CVD of developmental origins. Furthermore, thiosulfate may serve as a unique H_2_S donor. Thiosulfate can produce H_2_S through a nonenzymatic pathway or by an enzymatic pathway via a glutathione-dependent reduction [152]. On the other hand, H_2_S can be enzymatically oxidized in mitochondria to thiosulfate. Owing to thiosulfate having therapeutic potential in hypertensive animal models [153,154], there is an ongoing need for additional study to elucidate its reprogramming effects in cardiovascular programming.

### 5.3. Organosulfur Compounds

Organosulfur compounds derived from garlic or onions have drawn attention as natural precursors of H_2_S. Garlic-derived organic polysulfides have proved beneficial for attenuating hypertension-related disorders [155,156]. One possible reason is because garlic-derived polysulfides can mediate NO pathway, resulting in NO-mediated vasodilation [157]. Garlic-derived compounds also provide protection in atherosclerosis, diabetes, myocardial infarction, and ischemic stroke [158]. Little information currently exists with regard to their cardiometabolic health benefits in CVD of developmental origins. So far, only one report from our group demonstrated that supplementing garlic oil in pregnancy and lactation prevented hypertension programmed by a high-fat diet, which is associated with increased expression and activity of H_2_S-producing enzymes in offspring kidneys and reshaping gut microbiota [128].

Natural and synthetic isothiocyanates are also known as H2S donors [159]. Natural isothiocyanates are found in cruciferous vegetables such as broccoli, rocket, cauliflower, etc., [159]. Due to their beneficial biological effects, natural isothiocyanates have been exploited for the design of new synthetic analogs [160]. Broccoli consumption is associated with reduced risk of ischemic reperfusion injury-mediated cardiomyocyte death [161]. Another study demonstrated that 4-carboxyphenyl isothiocyanate processed cardioprotective properties in the ex vivo and in vivo I/R models [161]. Nevertheless, it is currently difficult to link isothiocyanates with cardiovascular programming because of the lack of data on their reprogramming effects.

### 5.4. Others

Numerous commonly used medications have been reported to increase H_2_S concentrations, such as metformin, amlodipine, ramipril, carvediol, atorvastatin, digoxin, aspirin, paracetamol, testosterone, vitamin D, and 17β-estradiol [152]. So far very few studies have targeted their potential in reprogramming with a focus on CVD, despite significant progress achieved in recent years on pharmacotherapies in the field of H_2_S research. Since many available H_2_S-releasing drugs are still in preclinical experiments, it would be interesting to see whether targeting the H_2_S downstream signal-related mechanisms would appear to be a practical approach to prevent CVD of developmental origins from further clinical translation.

Moreover, abundant SRB in the gut produce significant amounts of H_2_S and sulfur compounds. However, scant data have been reported on the effects of gut-derived H_2_S on cardiovascular programming [161]. More research on gut SRB and their products is required as they may become a potential therapeutic target in CVD of developmental origins.

However, a concern raised by these studies is that some H_2_S-based interventions have other actions beyond supplementing H_2_S production. For example, l-cysteine and NAC have antioxidant effects through the glutathione pathway; however, to what extent their reprogramming effects on CVD can be attributed to H2S deserves further elucidation. A better understanding of the H_2_S-dependent and -independent mechanisms responsible for the protective effects of various H_2_S-based interventions on CVD of developmental origins is therefore warranted.

## 6. Conclusions and Perspectives

The evidence supporting the potential therapeutic role of H_2_S-based therapy in CVD of developmental origins is robust but incomplete. This review has provided a general overview on the various H_2_S-based interventions that shows cardiovascular benefits, including precursors of H_2_S, H_2_S donors, and organosulfur compounds.

It stands to reason that early-life H_2_S-based interventions are considered as potential reprogramming therapies for CVD of developmental origins. However, it is noteworthy that H_2_S at supraphysiologic concentrations are toxic. Although some H_2_S-releasing agents (e.g., SG1002) have shown to be safe and well tolerated in Phase I clinical trials [7], attention will need to be paid to increase efficiency and reduce toxicity, and to be able to accurately monitor levels of H_2_S in vivo. Currently, various methods have been established for endogenous H_2_S measurement. Nevertheless, these methods have obvious limitations, especially in the clinical setting [127]. Therefore, future work in developing ideal methodology is needed to better assess H_2_S measurements in clinical practice and ensure H_2_S-based therapy would only apply in the case of deficits.

Meanwhile, we are aware that a long road still lies ahead in determining the right dose of H_2_S-based intervention for the right person, at the right time, for clinical applications. Little reliable information currently exists regarding the reprogramming effects of H_2_S-releasing drugs in human trials. Much of the preclinical work investigating the cardiovascular reprogramming actions of H_2_S has mainly studied hypertension. Since children with chronic kidney disease are at high risk for future CVD and they develop hypertension at early stages of CKD [162], and that early H_2_S-based interventions can prevent the transition of prehypertension to hypertension [93,136], there will be a growing need to examine their reprogramming effects by targeting other animal models of programmed and this specific study population.

Another important aspect is that significant progress has been made over the last few decades in H_2_S-releasing drugs, while less attention has been paid to gut bacteria-derived H_2_S. Lacking in the literature is how microbiota-targeted therapies may alter SRB to produce gut-derived H_2_S and whether it is beneficial or harmful for cardiovascular health.

Cardiovascular programming, apart from the H_2_S signaling pathway, has been linked to other common molecular mechanisms. What is the exact manner of cross-talk between these pathways? Are early-life interventions targeting other mechanisms (e.g., AMPK activator or RAS blockers) also able to mediate the H_2_S pathway to prevent CVD of developmental origins?

Finally, H_2_S is a meaningfully pathogenetic link for the developmental origins of CVD. After all this tremendous growth in H_2_S-based interventions and greater understanding of cardiovascular programming, we expect that H_2_S-based reprogramming therapies will be applied in clinics to reduce the global burden of CVD.

## Figures and Tables

**Table 1:** Summary of H_2_S-based modalities used as reprogramming interventions in animal models of cardiovascular programming.

H_2_S-Based Intervention	Animal Models	Species/ Gender	Age at Evaluation	Reprogramming Effects	Ref.
Precursors of H_2_S					
l-cysteine (8 mmol/kg/day) from 4 to 6 weeks of age	High-salt SHR	SHR/M	12 weeks	Prevented hypertension	[136]
D-cysteine (8 mmol/kg/day) from 4 to 6 weeks of age	High-salt SHR	SHR/M	12 weeks	Prevented hypertension	[136]
1% NAC in drinking water in pregnancy and lactation	Suramin administration	SD rat/M	12 weeks	Prevented hypertension	[57]
1% NAC in drinking water in pregnancy and lactation	Maternal L-NAME exposure	SD rat/M	12 weeks	Prevented hypertension	[76]
1% NAC in drinking water in pregnancy and lactation	Prenatal dexamethasone and postnatal high-fat diet	SD rat/M	12 weeks	Prevented hypertension	[62]
1% NAC in drinking water in pregnancy and lactation	Maternal hypertension	SHR rat/M	12 weeks	Prevented hypertension	[126]
NAC (500 mg/kg/day) in drinking water from gestational day 4 to postnatal day 10	Maternal nicotine exposure	SD rat/M	8 months	Prevented hypertension and myocardial ischemia-reperfusion injury	[137,138]
					
H_2_S donors					
NaHS (14 μmol/kg/day) daily intraperitoneal injection from 4 to 8 weeks of age	Genetic hypertension model	SHR/M	12 weeks	Prevented hypertension	[93]
NaHS (56 μmol/kg/day) daily intraperitoneal injection during pregnancy and lactation	2-kidney, 1-clip renovascular hypertension model	SD/M & F	16 weeks	Prevented hypertension and sympathetic activation	[139,140]
Organosulfur compounds					
Garlic oil (100 mg/kg/day) daily oral gavage during pregnancy and lactation	Maternal and postweaning high-fat diet	SD/M	16 weeks	Prevented hypertension	[128]

Studies tabulated according to types of intervention, animal models and age at evaluation. L−NAME = N^G^-nitro-L-arginine-methyl ester. M = male. F = female. NAC = N-acetylcysteine. NaHS = sodium hydrosulfide. SHR = spontaneously hypertensive rat. SD = Sprague−Dawley rat.
